# Supplementary material for: The burden of disabilities in Sidama National Regional State, Ethiopia: A cross-sectional, descriptive study
Source: PLoS One. 2023 Jul 19;18(7):e0288763. doi: 10.1371/journal.pone.0288763 (PMC10355417; doi:10.1371/journal.pone.0288763)
Supplement: S2 File — (DOCX) [file pone.0288763.s002.docx]

Supplementary file: S2 Questionnaires (English version)

8.2. English Version questionnaires

This questionnaire is designed to census reproductive age people with disabilities from the selected kebeles in Sidama Regional State, Ethiopia.

Wereda: ______________ Keble code___________ Household code ______________

| NO | Question | Response | Skip |
| --- | --- | --- | --- |
| **Section I: Disability, type and cause Census questions** | | | |
| 101 | Is there a person who has a problem with seeing, hearing, speaking and/or standing/walking/seating, body parts movement, functioning of hands/ legs or mental retardation, or a mental problem? | 1. Yes 2. No |  |
| 102 | If yes, what is the type of disability or problem? | 1. Both eyes blind: 1.Yes 2. No 2. Deaf: 1.Yes 2. No 3. Extremity paralysis or handicap: 1.Yes 2. No 4. Wheel-chaired/walking disability: 1.Yes 2. No 5. Confirmed mental disabilities:   1. Yes 2. No |  |
| 103 | What was the cause of disability? | 1. Fall 2. Burn 3. Poisoning 4. polio 5. Car Accident 6. Sharp objects 7. Farming equipment 8. Hit by another person by stick 9. Animal Bite 10. Inborn 11. Other (specify:________________) |  |
| 104 | Sex of the person | 1. Male 2. Female |  |
| 105 | The current age of the person | ____years ( in completed year) |  |
| 106 | What is the religion of the person? | 1. Orthodox 2. Catholic 3. Protestant 4. Muslim 5. Other (Specify)___________ |  |
| 107 | What is the ethnic group of the person? | 1. Sidama 2. Amhara 3. Oromo 4. Wolayita 5. Guragie 6. Others (specify)_____________ |  |
| 108 | What is the marital status of the person? | 1. Never married 2. Married 3. Divorced/separated 4. Widowed |  |
| 109 | Residence of the person | 1. Urban 2. Rural |  |
| 110 | What is the employment status of the person? | 1. Employed 2. Not employed |  |
| 111 | If ‘ employed; for Q 110, what is the type of employer | 1. Government 2. NGO 3. Private |  |
| 112 | What is the educational status of the person? | 1. Unable to read and write 2. Attended formal education |  |
| 113 | If attended formal education, specify the highest grade completed | ________Grade |  |
| 114 | Number of reproductive age (15-49 female and 15 and above male ) people in the household | Mention:__________________ |  |
| 115 | Total number of reproductive age (15-49 female and 15 and above male ) people in the Kebele (Ask from Kebele/woreda) | Mention:__________________ |  |
| 116 | The total population in the kebeles (Ask from Kebele/woreda) | Mention:__________________ |  |
