# Supplementary material for: The burden of disabilities in Sidama National Regional State, Ethiopia: A cross-sectional, descriptive study
Source: PLoS One. 2023 Jul 19;18(7):e0288763. doi: 10.1371/journal.pone.0288763 (PMC10355417; doi:10.1371/journal.pone.0288763)
Supplement: S3 File — (DOCX) [file pone.0288763.s003.docx]

Supplementary file:1S3 Questionnaires (Afo-sidamu)

**Qa’miso 8.2. SIDAAMU AFII GANO**

**Hawaasi Yuniverisite, Dagate Fayyimma Rosi Mine**

Aantete xa’minanni xa’muwa iillate diri meerero noo ama bisu aana xe’ne noorira calla dooraamino olliira Sidaamu Qoqqowi Mootimma giddo heedhanno teessaanora..

Worada: ______________ Olluu koodde___________ Minu koodde ______________

| A.k | Xa’mo | Dawaro | sai |  |
| --- | --- | --- | --- | --- |
| **Gafa I: Hasiisote bisu xe’ne ikkito, hiittennenna may koorkaatinniiti bisu xe’ne xaaddinoro buunxanni xa’muwa** | | | |  |
| 101 | La’’ate qarri/maciishshate qarri/coyi’rate qarri/lekkate uurre ha’rate qarri/ofolla hoogate qarri/bisu millimo hoggate qarri/angate hoonge qarri/buqqeete xe’ne qarri/mannimmate xe’ne qarri noohu no? | 1. Ee 2. Dee’ni |  |  |
| 102 | Xa’mo 101 dawarokki Ee, ikkiro, hiitti bisu xe’ne noohe? | 1. Lamenti ille dila’anno: 1.Ee 2. Dee’ni 2. Macciishsha didadiitanno.: 1.Ee   Dee’ni   1. Lekka woy anga laanshawate: 1.Ee 2. Dee’ni 2. Uuritte hadhe dandiiktannokite: 1.Ee 2. Dee’ni 3. Buuxantinoti buqqeete xisso noote:   1. Ee 2. Dee’ni |  |  |
| 103 | Bisu xe’ne abbihehu maati? | 1. Uwatenniiti. 2. Giirate 3. Haadho agatenni. 4. poliyootenni 5. Kaameelu danooti. 6. Qara afi’rinorichooti. 7. Baatto loosi’nanni uduunninniiti. 8. Mannu anganni bordunni ganameeti. 9. Saada ga’mitinoe 10. Bisu xe’nenni hee’re ilamoomma. 11. Wole xawisi (:________________) |  |  |
| 104 | Koo/tee | 1. Koo 2. Tee |  |  |
| 105 | Xa dirikki me’’eho? | ________dirooti. |  |  |
| 106 | Amma’nokki hiittenneeti? | 1. Ortodokise 2. Kaatoolikete 3. Protesitaante 4. Islaamaho 5. Wole xawisi ___________ |  |  |
| 107 | Hiittenne daga giddo gaamamatta? | 1. Sidaamaho 2. Amaaraho 3. Oromote 4. Wolayitta 5. Guraage 6. Wole xawisi_____________ |  |  |
| 108 | Adhammekki dani? | 1. Leexa 2. Adhaminoha 3. Adhe/ ite Tirroha 4. Baxxinoha |  |  |
| 109 | Teessokki? | 1. Quchumaho 2. Baadiyyete |  |  |
| 110 | Loosikki qeechi? | 1. Mootimmate loosaasincho. 2. Mottimmate loosaasincho ikkitinokkite |  |  |
| 111 | Xa’mo 110 umikki doorsha ikkiro, hiikkonne looso? | 1. Mootimmate looso 2. Mootimmanniha ikkinokki looso 3. Hallanyu looso |  |  |
| 112 | Rosikki deerri? | 1. Nabbawanna borreessa didandiintanno 2. Xaddote roso rosinoho. |  |  |
| 113 | Xamo 112 layinki doorsha ikkiro, me’’e kifile geeshsha rosootta? | ________kifile |  |  |
| 114 | Meyaa beetto 15-49 diri mereero noo kiironna 15 diri ali labbaaha mini’ne giddo noore kiirootenni fooqa darga wori. | Kiirotenni_____________ |  |  |
| 115 | Meyaa beetto 15-49 diri mereero noo kiironna 15 diri ali labbaha ollii’nera (ollaa woy worada xa’mi) | kiirotenni:__________________ |  |  |
| 116 | Xaphoomunni olluu giddo mageeshshi manni kiiro nooro fooqa darga wori.(olluu manna woradu manna xa’mi) | kiirotenni:__________________ |  |  |
