# Supplementary material for: The burden of disabilities in Sidama National Regional State, Ethiopia: A cross-sectional, descriptive study
Source: PLoS One. 2023 Jul 19;18(7):e0288763. doi: 10.1371/journal.pone.0288763 (PMC10355417; doi:10.1371/journal.pone.0288763)
Supplement: Attachment — Submitted filename: Comments for PLOS One_The Burden of Disabilities in Sidama National Regional State Ethiopia.docx [file pone.0288763.s005.docx]

The aticle titled “The Burden of Disabilities in Sidama National Regional State, Ethiopia: A Cross-Sectional, Descriptive Study” has been reviewed. Overall, this is an interesting study while the data will be benefeicial for the policy maker as it use a large sample size. However, there are some issues need to be clarified as follows:

1. In the background, the authors stated the prevalence of disabilities were varied across the countries. It may sound normally as the various character found in each country may resulted in different prevalence. Then, why did the author stated this reason to identify the significance of study? As this is an original study (not the meta-analysis), the results may showed a prevalence that may be different from others again. The gap analysis should be more clear and described in this section.
2. How was the calculation of sample size? What is the reference used to calculate it? Is it based on the previous study or using some statistical analysis such as G power analysis.
3. In the data collection procedure, the authors mentioned “…gathering the community in the kebele’s venue”. What does it mean here? What was the purpose of this procedure?
4. The table 1, the authors provide the data of “widowed” and “divorced”. It may overestimates as widowed may be one type of divorced. Please use more clear and sharp terminology. In addition, the same case with the term “non-government” and “private”. These may cause bias. The
5. Table 2, why did the authors categorize the prevalence of disability into several age category? Is there any reference showing the significance different among those categories? In addition, if so, please add it on the research aim.
6. In the discussion section, the authors described that the exclusion of under-five years participants may cause the difference finding. Please add the reference why the under-five years participants may contribute to the difference. Is there any study supported?
7. In the discussion section, paragraph 6, “The other important finding of this study is assessing the status of disability associations and membership of disabilities”. What does the meaning of disability association? Why is it important?
8. Please do a proof read to ensure each sentence provide clear and sharp meaning.
