# Supplementary material for: The burden of disabilities in Sidama National Regional State, Ethiopia: A cross-sectional, descriptive study
Source: PLoS One. 2023 Jul 19;18(7):e0288763. doi: 10.1371/journal.pone.0288763 (PMC10355417; doi:10.1371/journal.pone.0288763)
Supplement: Attachment — Submitted filename: Rebuttal Letter .docx [file pone.0288763.s006.docx]

**Date: 30/06/2023**

**Response letter**

Dear editors

We would like to thank the reviewers and the editor for their invaluable comments, suggestions and their time.

We addressed all the issues raised by the reviewers and editor using their questions and responses, using blue for each comment and question one by one.

We believe the manuscript has been modified based on the given comments and is suitable for publication in PLOS ONE journal.

Zelalem Tenaw (Assistant Professor of Maternity and Rh)

On behalf of all authors

**Editor’s comments (additional requirements) and responses**

1. Please ensure that your manuscript meets PLOS ONE's style requirements, including those for file naming.

**Response:** Dear editor, thank you very much. The manuscript meets PLOS ONE’s style.

2. In the ethics statement in the Methods, you have specified that verbal consent was obtained. Please provide additional details regarding how this consent was documented and witnessed, and state whether this was approved by the IRB.

**Response:** The study was approved by the Hawassa University IRB. Then the head of the household asked his or her voluntariness; if volunteer, we tick “yes” and continue; if not volunteer, we tick “no” and leave the household. This was witnessed in the presence of the kebele leader (lines 149-151).

3. Please include a separate caption for each figure in your manuscript.

**Response:** Thank you for the comment. We included a separate caption for both figures.

4. Please include captions for your Supporting Information files at the end of your manuscript, and update any in-text citations to match accordingly.

**Response:** Thank you again for the comment. We included a caption for both the supporting information.

5. Please review your reference list to ensure that it is complete and correct. If you have cited papers that have been retracted, please include the rationale for doing so in the manuscript text, or remove these references and replace them with relevant current references. Any changes to the reference list should be mentioned in the rebuttal letter that accompanies your revised manuscript. If you need to cite a retracted article, indicate the article’s retracted status in the References list and also include a citation and full reference for the retraction notice.

**Response:** Thank you for the comments. We reviewed our references and ensured they are complete and correct. And also, we have not cited retracted papers.

**Reviewer 1 comments and responses**

1. In the background, the authors stated the prevalence of disabilities were varied across the countries. It may sound normally as the various character found in each country may resulted in different prevalence. Then, why did the author stated this reason to identify the significance of study? As this is an original study (not the meta-analysis), the results may show a prevalence that may be different from others again. The gap analysis should be clearer and more described in this section.

**Response:** Dear reviewer, thank you for the very constructive comment. We modified the ambiguous statement (see lines 60-62). Otherwise, the gap is clearly showed (see lines 74-81).

2. How was the calculation of sample size? What is the reference used to calculate it? Is it based on the previous study or using some statistical analysis such as G power analysis.

**Response:** Thank you again for your comment. We selected 30 kebeles from 66 kebeles based on the estimated number of people with disabilities that we used for the other components of this projects, like (1). After randomly selecting the kebeles, we totally censused all the households present in the selected 30 kebeles (20 rural and 10 urban).

3. In the data collection procedure, the authors mentioned “…gathering the community in the kebele’s venue”. What does it mean here? What was the purpose of this procedure?

**Response:** Dear reviewer, thank you very much for your observation and comment. We modified the terminology gathering to assembling (see line 114). The purpose of the assembling was done to minimize the informational bias of the head or leader of the household (see lines 115-116).

4. The table 1, the authors provide the data of “widowed” and “divorced”. It may overestimate as widowed may be one type of divorced. Please use more clear and sharp terminology. In addition, the same case with the term “non-government” and “private”. These may cause bias.

**Response:** Thank you again. In our context, widowed has totally different meaning from divorced. Therefore, we believe that it is better to maintain the category as it is. Regarding types of employers, we removed it to minimize confusion.

5. Table 2, why did the authors categorize the prevalence of disability into several age category? Is there any reference showing the significance different among those categories? In addition, if so, please add it on the research aim.

**Response:** Thank you for your comment and concern. We believed that, since the study is descriptive, showing the burden of disability by age category had many importance for different stockholders to design appropriate strategies based on the age categories and it provides additional information

6. In the discussion section, the authors described that the exclusion of under-five years participants may cause the difference finding. Please add the reference because the under-five years participants may contribute to the difference. Is there any study supported?

**Response:** Thank you for your observation and comment. The evidence is supported with reference number 10. The justification is that, when the under-five included in the denominator, the prevalence decreased and prevalence roses with age (2).

7. In the discussion section, paragraph 6, “The other important finding of this study is assessing the status of disability associations and membership of disabilities”. What does the meaning of disability association? Why is it important?

**Response:** Dear reviewer, Thank you again for your comment. Disabilities associations are one of the important associations to reach and support people with disabilities. That is why assessing disabilities association membership is important.

8. Please do a proof read to ensure each sentence provide clear and sharp meaning.

**Response:** Thank you very much your comment. We did a proof reading to improve the language problems of the manuscript.

1.	Tenaw Z, Gari T, Gebretsadik A. Contraceptive use among reproductive-age females with disabilities in central Sidama National Regional State, Ethiopia: a multilevel analysis. PeerJ. 2023;11:e15354.

2.	Tamrat YK, Shitaye Alemu, John Moore, Girmaye,. The prevalence and characteristics of physical and sensory disabilities in Northern Ethiopia. journal of disability and rehabilitation. 2001;23(17):799-804.
